# Integration of Nascent Transcriptomes and Cross-Species Atlases Reveals Evolutionary Barriers in Transcriptional Kinetics During Mammalian Hibernation

**DOI:** 10.3390/vetsci13101062

**Published:** 2026-10-09

**Authors:** Zhuopeng Hu, Shidi Gao, Yilin Wu, Yifan Wang, Lei Yu, Huimei Yu

**Affiliations:** 1 Department of Pathophysiology, the Medical Basic Research Innovation Center of Airway Disease in North China, Key Laboratory of Pathobiology, Ministry of Education, College of Basic Medical Sciences, Jilin University, Changchun 130021, China; huzp9923@mails.jlu.edu.cn (Z.H.); gaosd26@mails.jlu.edu.cn (S.G.); wuyl9922@mails.jlu.edu.cn (Y.W.); wangyf8222@mails.jlu.edu.cn (Y.W.); 2 Department of Physiology, the Medical Basic Research Innovation Center of Airway Disease in North China, Key Laboratory of Pathobiology, Ministry of Education, College of Basic Medical Sciences, Jilin University, Changchun 130021, China; y_lei@jlu.edu.cn

**Keywords:** hibernation, transcriptional kinetics, cross-species multi-omics, network topology, metabolic remodeling, post-transcriptional regulation

## Abstract

Mammalian hibernation requires extensive metabolic remodeling, but traditional transcriptomic approaches cannot distinguish between mRNA synthesis and degradation, leaving the evolutionary barriers governing its transcriptional control unclear. To address this, we integrated nascent (GRO-seq) and steady-state (RNA-seq) transcriptomes from the 13-lined ground squirrel *(Ictidomys tridecemlineatus)* with multi-species liver data from the Siberian chipmunk and Syrian hamster. By constructing an atlas of 78 core consensus genes and applying the stability index (SI), betweenness centrality (BC), and network clustering, we identified two complementary regulatory modes: a Triple Conserved backbone centered on ACLY and ATF4, alongside “Species-Specific Synergy” modules involving ADCY6 and EGR1. Kinetic analysis revealed distinct transcriptional-decoupling patterns (SLC2A1 showed a transcript stabilization pattern with SI = 2.01, whereas ID2 exhibited an uncompensated transcriptional induction with SI = −1.87). Network analysis placed ADCY6 (BC = 0.3356) as a central hub linking signal transduction to metabolic remodeling, and MCODE clustering further resolved three functional modules enriched for metabolic regulation, RNA processing, and lipid homeostasis. Together, these results suggest that hibernation does not simply suppress transcription but instead operates as a dynamic balance system, where specific genes rely on post-transcriptional stability compensation under sustained transcriptional stress. These findings offer a cross-species view of how mammals achieve metabolic robustness and may inform cryopreservation strategies in translational contexts.

## Simple Summary

Hibernation is an important but often misunderstood survival strategy that allows some mammals to endure extreme cold. However, how cells control gene activity during this state remains poorly understood. In this study, we examined liver cells from three different hibernating rodents to uncover the molecular strategies behind this process. We found that rather than simply shutting down, these animals manage their genes through two distinct evolutionary strategies: a set of triple conserved genes is consistently regulated across all three species, forming a shared survival foundation, while other genes follow species-specific synergy patterns that reflect different evolutionary paths. We also identified certain genes that act as central control hubs, coordinating how cells manage energy and maintain stability in the cold. This article provides readers with insights into the active and finely tuned nature of hibernation, which may help to improve our understanding of how mammals adapt to extreme environments.

## 1. Introduction

Mammalian hibernation is a precisely orchestrated systemic physiological transition involving extreme thermoregulation, metabolic suppression, and deep remodeling of cellular functions [1,2]. Although transcriptomic studies over the past decade have identified thousands of differentially expressed genes, traditional steady-state transcriptome (RNA-seq) analysis often only captures the final static result of the intracellular mRNA pool [3]. It overlooks the complex dynamic balance between transcriptional synthesis and mRNA stability, as the total pool of mRNA often remains constant throughout torpor despite global reductions in energy-expensive processes like transcription [4,5,6]. Recent studies have begun utilizing nascent RNA sequencing, such as Global Run-On sequencing (GRO-seq), to demonstrate that while seasonal metabolic reprogramming is primarily transcriptionally driven, the torpor-arousal cycle is shaped by a shifting balance between real-time transcription and RNA stability [6]. This lack of information makes it difficult to distinguish which genes are real-time drivers of hibernation onset and which are mRNA transcripts retained to sustain translation under prolonged cold exposure to cope with long-term cold, a challenge further highlighted by proteomic studies revealing major differences between steady-state mRNA and actual protein expression levels during torpor [7,8]. More importantly, on an evolutionary scale, whether hibernating species from different Orders share a common molecular “blueprint” or have evolved divergent yet synergistic survival strategies remains a core scientific question in the field of hypometabolism [9,10,11].

To deconstruct this complex regulatory landscape, this study integrates GRO-seq and steady-state transcriptome data from the 13-lined ground squirrel (*Ictidomys tridecemlineatus*). We further link these with the Siberian chipmunk (*Eutamias sibiricus*) and Syrian hamster (*Mesocricetus auratus*), which share similar mechanisms but independent evolutionary paths, to construct a comprehensive integrated atlas of 78 core regulatory factors. We introduce a novel approach by proposing the Stability Index (SI) to assess the degree of decoupling between nascent transcription and mRNA steady-state levels. Our findings reveal that hibernation is not a simple genome-wide silence, but a dynamic process involving transcriptional bursts and precise degradation. Through system biology topological analysis, we identified a series of high-Betweenness-Centrality hub nodes, represented by adenylate cyclase ADCY6 and phospholipid phosphatase PLPP3 [12,13]. These nodes serve as key intermediaries between signal transduction and lipid metabolism remodeling, exhibiting systemic weights in the network that far exceed their fold-change in expression.

The data further reveal binary characteristics of hibernation strategies in evolution. On one hand, Triple Conserved genes, represented by ATP citrate lyase (ACLY) and activating transcription factor 4 (ATF4), form the fundamental molecular backbone of hibernation, reflecting the convergent evolutionary trend of distantly related species when facing extreme energy scarcity. This molecular “toolkit” for dormancy appears evolutionarily ancient, as immediate early genes like early growth response 1 (EGR1) have been identified as key regulatory signatures in the torpor states of species ranging from colonial urochordates [14] to sea cucumbers [15], suggesting a conserved mechanism for environmental adaptation. This is further supported by the recent identification of conserved noncoding cis-regulatory elements (CREs) that modulate metabolic and behavioral adaptations across diverse hibernating lineages. On the other hand, we observed significant phase differences between nascent transcriptional activity and steady-state abundance for key factors such as EGR1 and Solute carrier family 2 member 1 (SLC2A1) [16]. This decoupling of transcriptional kinetics reveals a selective transcriptional regulatory mechanism used by hibernating animals to maintain intracellular homeostasis at extremely low body temperatures [6], in which non-coding RNAs and microRNAs frequently act as key executors of post-transcriptional suppression during metabolic rate depression [17,18]. While previous work by Gillen et al. [6] mapped transcriptional kinetics in the ground squirrel alone, the evolutionary conservation of these decoupling events across diverse lineages remains uncharacterized. Therefore, the objective of this study is to advance beyond single-species studies by characterizing the conserved and species-specific transcriptional regulation mechanisms across three distinct mammalian hibernators. By analyzing shared pathways and integrating topological kinetics, this study helps explain the basis of hibernation conservation at the molecular level and provides an evolutionary context for understanding metabolic adaptation under extreme stress.

## 2. Methods

### 2.1. Data Acquisition and Multi-Species Integration Framework

To construct a high-resolution molecular landscape of the hibernating liver, this study established an analytical framework integrating four high-throughput sequencing datasets. These cover three key rodent lineages: the Siberian chipmunk, the 13-lined ground squirrel, and the Syrian hamster. This multi-species strategy aims to identify conserved regulatory features of mammalian responses to hypometabolic stress, spanning approximately 100 million years of evolutionary distance through comparative genomics analysis.

### 2.2. Proprietary Transcriptomic Profiling of E. sibiricus

Liver transcriptomic data for the Siberian chipmunk were generated by our laboratory. Samples were collected from 6 adult individuals, divided into two strictly defined physiological groups: the normothermic control group (Control, *n* = 3; kept under room-temperature conditions (23 ± 2) °C, core body temperature 37 °C) and the deep hibernation group (Hibernation, *n* = 3; kept in a constant-temperature box at (4 ± 1) °C without light, maintained in continuous torpor for at least 3 days with core body temperature 5–8 °C). Total RNA was isolated using the oligo(dT) magnetic bead enrichment method, followed by library construction using a strand-specific protocol. Sequencing was performed on the Illumina platform (NEB, Ipswich, MA, USA) in PE150 mode, generating over 40 Gb of Clean Data with a Q30 quality score ≥ 96.21%. Clean reads were aligned to the reference genome (GCA 025594165.1) using HISAT2 (v2.1.0), achieving an alignment efficiency of 83.14%. Gene-level quantification and transcript assembly were completed using StringTie (v1.3.3).

### 2.3. Public Genomic Resource Integration

To supplement the proprietary data, multimodal genomic resources were retrieved from the NCBI Gene Expression Omnibus (GEO). For the ground squirrel, a transcriptional kinetics model was constructed by integrating steady-state transcriptomes (RNA-seq, GSE166814; summer active (SA) *n* = 5, late torpor (LT) *n* = 5) and nascent transcriptomes (GRO-seq, GSE166370; SA *n* = 3, LT *n* = 3) from raw count matrices [6]. For the Syrian hamster, a genome-wide read count matrix was obtained from GSE199814 (SA *n* = 3, torpor *n* = 3) [19]. For cross-species ortholog mapping, a standardized programmatic pipeline was established. For the Siberian chipmunk, orthologous Gene Symbols were precisely extracted from Swiss-Prot annotations using regular expression matching (regex GN=). For the Syrian hamster, Ensembl Gene IDs were converted to human ortholog Gene Symbols via the mygene Python library (taxon: 10036; v3.2.2; https://pypi.org/project/mygene/, accessed on 25 September 2026). Subsequently, cross-species integration was performed using a strict inner join (intersection) across all aligned datasets, ensuring that downstream analyses were restricted to genes unambiguously identified in all three species.

### 2.4. Quantification of Transcriptional Kinetics

The raw read count matrices for both RNA-seq and GRO-seq were processed using the DESeq2 statistical framework (implemented via PyDESeq2 v0.44.0 in Python 3.9.13; https://pydeseq2.readthedocs.io/, accessed on 25 September 2026). This approach utilizes negative binomial generalized linear models to account for biological variance and library size differences. For all datasets (chipmunk, ground squirrel, and hamster), statistical significance was determined using the Wald test, followed by multiple-testing correction via the Benjamini–Hochberg procedure. A False Discovery Rate (FDR/*p*-adj) < 0.05 was adopted as the uniform threshold for defining statistically significant differential expression. Prior to DESeq2 fitting, low-expression genes (total cross-sample counts < 10) were filtered out. Furthermore, potential sample outliers were automatically flagged and handled using the built-in Cook’s distance metric within the PyDESeq2 framework.

For the dual-omics data of the ground squirrel, we defined the SI: SI = log2FC_RNA − log2FC_GRO. This index is used to distinguish genes driven by real-time transcriptional synthesis from those driven by post-transcriptional stability regulation.

### 2.5. PPI Modeling and Topological Prioritization

Based on the 78 core hibernation-associated genes, we constructed a functional association network using the STRING database (Version 12.0; https://string-db.org/, accessed on 25 September 2026). To ensure maximum interactome coverage across non-model rodent species, orthologous Gene Symbols were mapped against the well-annotated Homo sapiens reference background. The network was generated using the “full STRING network” option to capture both functional and physical associations, with edges representing interaction evidence. All active interaction sources were enabled (Textmining, Experiments, Databases, Co-expression, Neighborhood, Gene Fusion, and Co-occurrence), and the minimum required interaction score was set to medium confidence (>0.400).

The network was modeled as a weighted graph using the Python NetworkX library (v2.8; https://networkx.org/, accessed on 25 September 2026). Rather than using generic physical interaction probabilities, we weighted the edges based on the absolute deviation in transcriptional kinetics between connected nodes (Wi,j = |ΔSIi,j|). Because interacting proteins in functional modules require stoichiometric coordination, nodes connecting partners with highly asynchronous transcriptional kinetics face greater regulatory divergence. Consequently, by incorporating this stability deviation as an edge weight, the Betweenness Centrality (BC) calculation accurately quantifies a node’s importance in linking transcriptionally uncoupled pathways across the systemic hibernation network. Rather than testing against a random null-hypothesis network, BC was strictly utilized in this study as an empirical, relative ranking metric to prioritize structural bottlenecks within our specific 78-gene functional interactome.

### 2.6. MCODE Clustering and Visual Mapping Standards

To identify highly synergistic functional modules within the network, the Molecular Complex Detection (MCODE) plugin was executed within the Cytoscape (v3.9.1) environment. Clustering parameters were set to standard stringent thresholds (Degree Cutoff = 2, Node Score Cutoff = 0.2, Haircut = True, and K-Core = 2) to ensure reproducibility and to prioritize tightly interconnected protein networks over loosely connected modules. Since MCODE assigns a deterministic cluster score based on local network density, clusters with a score ≥ 2.0 were considered biologically meaningful for downstream pathway analysis.

## 3. Results

### 3.1. Identification of the Molecular Backbone and Evolutionary Divergence in Mammalian Hibernation via Cross-Species Integration

To identify the shared regulatory features of hibernation at a systems level, we integrated transcriptomic data from the ground squirrel, chipmunk, and Syrian hamster following DESeq2 statistical modeling. The core integrated atlas of 78 high-confidence genes (Figure S1, Supplementary Table S1) was anchored by the functionally annotated hibernation signatures from our proprietary Siberian chipmunk dataset. We avoided forcing an arbitrary differential expression threshold (e.g., FDR < 0.05) across all three highly divergent species. Such a rigid threshold would artificially eliminate genes undergoing compensatory stabilization where steady-state mRNA levels remain strictly unchanged. Instead, we defined this core atlas by ensuring strict 1:1 ortholog mapping across the ground squirrel, chipmunk, and hamster. This robust intersection approach allowed us to track the diverse kinetic behaviors (SI) of these 78 essential hibernation-associated genes across different evolutionary lineages, regardless of their steady-state statistical significance in any single species. Furthermore, STRING database analysis confirmed that this 78-gene consensus set exhibits a highly significant protein–protein interaction (PPI) enrichment (154 observed edges vs. 50 expected edges; PPI enrichment *p*-value < 1.0 ×10^−16^), demonstrating that these cross-species mapped genes form a significant functional interaction network. Within this atlas, candidates were quantitatively classified into two evolutionary strategies based on the mathematical alignment of their expression directionality (sign of log2FC) across all three species. Genes were defined as Triple Conserved if and only if their differential expression polarity was strictly identical across the three species; otherwise, they were categorized as Species-Specific Synergy.

**Figure 1:**
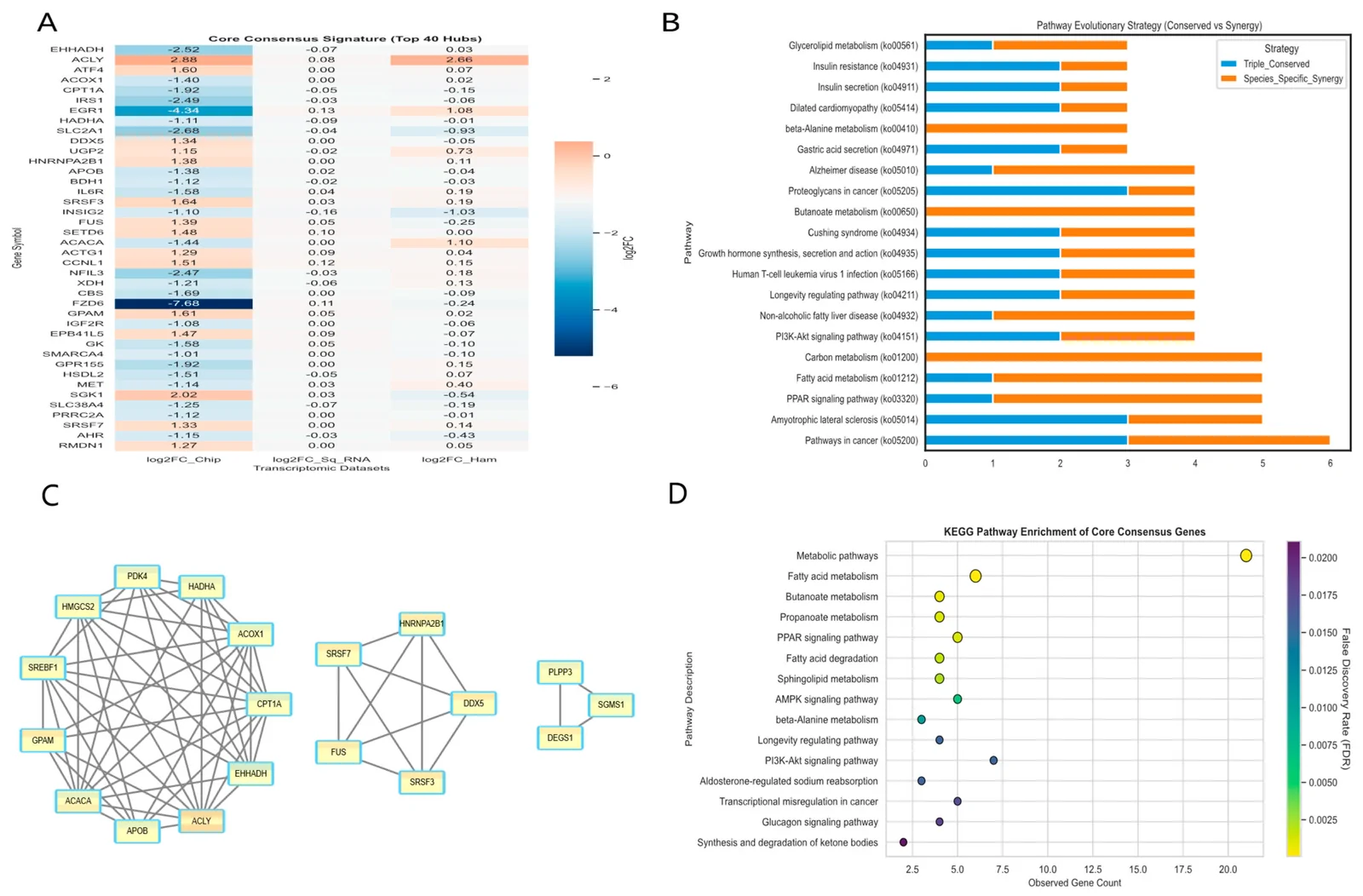
Comprehensive transcriptional landscape and kinetic architecture of the hepatic hibernation program. (**A**) Consensus transcriptional signature of the hepatic hibernation program. Heatmap displaying the log2-transformed fold changes (log2FC) of the 40 most significant genes consistently regulated across Eutamias sibiricus, Ictidomys tridecemlineatus, and Mesocricetus auratus. Genes are ranked by False Discovery Rate (FDR). Each cell represents the population-level average, identifying a core molecular signature for hepatic survival during torpor, anchored by ACLY and CPT1A. (**B**) Pathway-level distribution of evolutionary strategies. Stacked bar chart quantifying the ratio of Triple Conserved (Blue) versus Species-Specific Synergy (Orange) strategies across the top 20 enriched KEGG pathways. Fundamental modules, such as the Spliceosome, are dominated by conservation, while complex metabolic rewiring is primarily driven by species-specific synergistic adaptation. (**C**) MCODE-based identification of functional execution units. Modular decomposition of the 78-gene network into three high-density clusters. Cluster 1 (Metabolic Engine, Score = 6.8): Orchestrates the transition from glucose to lipid utilization (ACLY, CPT1A). Cluster 2 (Information Preservation, Score = 4.0): Focuses on RNA processing and splicing machinery maintenance (SRSF3, DDX5). Cluster 3 (Membrane Homeostasis, Score = 2.0): Mediates cold-adaptive lipid and sphingolipid remodeling (DEGS1, SGMS1). Nodes are annotated with Stress scores to denote their role as systemic communication bottlenecks. (**D**) Functional enrichment of the hibernation core. KEGG pathway enrichment dot plot showcasing the top globally enriched metabolic and signaling pathways corresponding to the functional clusters. Bubble size represents the observed gene count, and color intensity scales with the false discovery rate (FDR).

Analysis showed that genes with Triple Conserved characteristics form the core basis of hibernation physiology. Among them, ACLY (ATP citrate lyase) exhibited a strong cross-species upregulation (e.g., Chipmunk log2FC = 2.88, FDR < 0.001; Sq_RNA log2FC = 0.08, FDR < 0.001; Hamster log2FC = 0.07, FDR = 0.046). Simultaneously, ATF4 (a key factor in the integrated stress response) and HK2 (a rate-limiting enzyme in glucose metabolism) showed synergistic upregulation (Figure 1A).

However, quantitative pathway evolutionary statistics revealed significant heterogeneity in metabolic paths (Figure 1B, Supplementary Table S2). Although fatty acid metabolism and carbon metabolism were significantly regulated in all studied species, their constituent genes mostly exhibited a “Species-Specific Synergy” pattern (e.g., 4 out of 5 core regulators in Carbon Metabolism displayed species-specific plasticity, including ground squirrel-specific induction of EHHADH and hamster-specific induction of LIPG). In contrast, pathways maintaining basic cellular functions, such as the Spliceosome, showed extremely high conservation, with all differentially expressed members consistently following the Triple Conserved pattern.

A notable finding was the high divergence in transcriptional polarity. Taking EGR1 as an example, nascent transcription (GRO-seq) in the ground squirrel showed an extremely active transcriptional burst (log2FC = 4.42, FDR < 0.001), but steady-state levels in the chipmunk showed a severe downregulation (log2FC = −4.34, FDR < 0.001).

### 3.2. Transcriptional Kinetic Analysis Reveals Deep Decoupling Between Steady-State Expression and Synthetic Activity

Steady-state mRNA levels are often used as direct indicators of gene activity. However, by integrating the nascent (GRO-seq) and steady-state (RNA-seq) transcriptomes of the ground squirrel, we observed a detailed pattern of transcriptional kinetics. We quantified the deviation between synthesis rates and abundance by calculating the Stability Index (SI = log2FC_RNA − log2FC_GRO). To rigorously define transcriptional decoupling, we evaluated the statistical significance of both components using DESeq2 (FDR < 0.05). The results indicated that a substantial portion of core genes exhibited significant kinetic decoupling during hibernation, revealing a systemic regulatory strategy rather than isolated events (Supplementary Table S1).

In this atlas, SLC2A1 (glucose transporter GLUT1) exhibited the most typical compensatory stabilization decoupling pattern. Its nascent transcriptional activity underwent a significant downregulation during hibernation (log2FC_GRO = −2.05, FDR < 0.001), yet its steady-state mRNA abundance was actively maintained without significant reduction (log2FC_RNA = −0.04, FDR = 1.0), resulting in an SI of 2.01. Similar statistically validated phenomena were observed in CBS (cystathionine beta-synthase, SI = 1.27, GRO-seq FDR = 0.004) and ACTG1 (SI = 1.68, GRO-seq FDR < 0.001). This hypothesis was further validated in subsequent network clustering analysis: MCODE Cluster 2 (Information Preservation module) integrated many core factors maintaining RNA stability, including SRSF3 and DDX5, supporting its role in selective post-transcriptional stabilization (Figure 1C).

**Figure 2:**
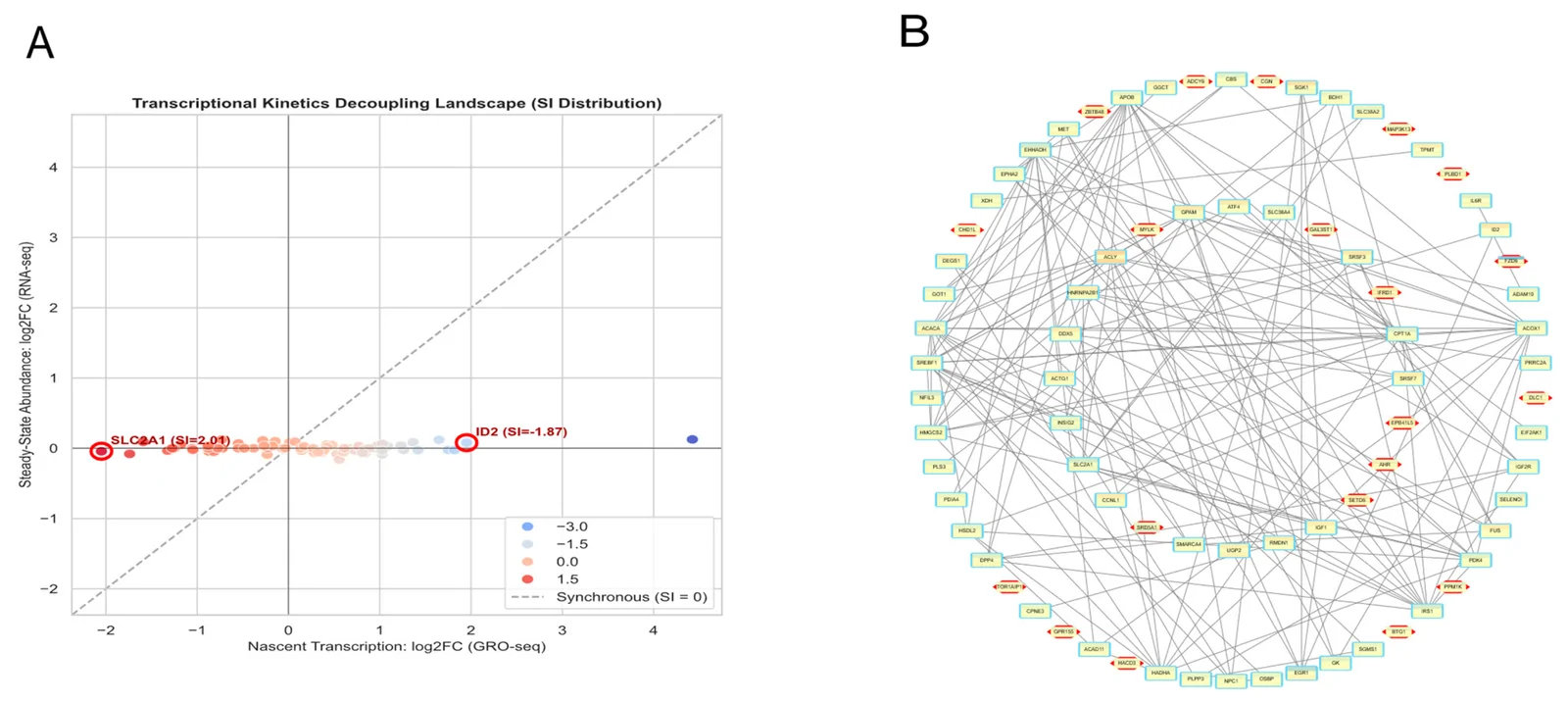
Macroscopic kinetic decoupling and microscopic topological architecture. (**A**) Global kinetic decoupling landscape based on the Stability Index (SI = log2FC_RNA − log2FC_GRO). The scatter plot illustrates systemic decoupling events across the 78-gene core atlas. Hexagonal nodes highlight extreme kinetic behaviors, including the compensatory stabilization pattern (SLC2A1, SI = 2.01) and the uncompensated transcriptional induction pattern (ID2, SI = −1.87). (**B**) Integrated regulatory topology visualized using a Nested Concentric Circle layout. The inner ring isolates Triple Conserved evolutionary anchors, while the outer ring represents Species-Specific Synergy genes. The network architecture identifies ADCY6 as a central hub (Betweenness Centrality = 0.3356), linking functionally decoupled nodes.

Conversely, another group of key regulators exhibited an uncompensated transcriptional induction pattern represented by ID2 (inhibitor of DNA binding 2) and PDK4 (pyruvate dehydrogenase kinase 4). Data showed that while the nascent transcriptional activity of ID2 jumped massively during hibernation induction (log2FC_GRO = 1.95, FDR < 0.001), its steady-state mRNA pool did not accumulate synchronously (log2FC_RNA = 0.08, FDR = 1.0), resulting in a highly negative Stability Index (SI = −1.87). Functional clustering analysis of genes with extreme Stability Indices found that this kinetic decoupling was highly enriched in fatty acid oxidation (e.g., CPT1A, SI = 0.82, GRO-seq FDR = 0.009) and signal transduction pathways (Figure 2A).

### 3.3. Network Topology Mining Identifies Core Evolutionary Hubs Driving Hibernation Phenotype Transitions

To identify key factors acting as master regulators in the complex molecular network of hibernation, we constructed a cross-species functional association network based on shared KEGG pathway interactions and introduced the Betweenness Centrality (BC) algorithm (Figure 2B and Figure S2). Unlike traditional fold-change analysis, the BC score quantifies a gene’s ability to act as a “bridge” connecting different biological functional modules. The results showed that the network exhibits significant scale-symmetry, with a few gene entities possessing high BC scores dominating the steady-state hibernation landscape.

Among all integrated genes, adenylate cyclase ADCY6 (BC = 0.3356) showed the highest topological significance. Although its steady-state expression levels changed relatively modestly across species, it connected more than 50 key pathways in the network, including the cAMP signaling pathway, thermogenesis, and various neurotransmitter synapses. Another notable topological hub is the phospholipid phosphatase PLPP3 (BC = 0.318). This gene plays the role of a “metabolic intersection” in the network, successfully linking multiple lipid regulatory modules such as glycerophospholipid metabolism, sphingolipid signaling, and phospholipase D signaling. The systemic downregulation of PLPP3 was notably observed in the chipmunk (log2FC = −1.18) and hamster (log2FC = −1.23). Additionally, top-ranked hubs in betweenness centrality included CPT1A (BC = 0.270) and SLC2A1 (BC = 0.235), which act as gates for fatty acid and glucose transport, respectively, deeply coupling energy substrate preference shifts with intracellular signal flow. Notably, among the Top 10 core hub genes identified by network topology analysis (e.g., EGR1, EHHADH, APOB), more than 70% belong to the “Species-Specific Synergy” category.

### 3.4. MCODE Cluster Analysis Identifies Core Functional Modules of Hibernation Regulation

To further reveal the topological structure of the integrated hibernation molecular network, we utilized the MCODE algorithm for sub-network mining. The analysis identified three functional clusters with significant biological relevance, representing the synergistic operational units of different physiological processes during hibernation.

Cluster 1 (MCODE Score = 6.8) exhibited the highest clustering density, comprising core metabolic enzymes such as ACLY, CPT1A, HADHA, and EHHADH. This module is significantly enriched in carbon metabolism, the TCA cycle, and fatty acid degradation pathways (KEGG enrichment FDR < 0.01, Figure 1D, Supplementary Table S3), acting as a core pathway for the transition from glucose to lipid substrates during hibernation. Notably, genes within this module, such as CPT1A and ACACA, possess extremely high Stress scores (708 and 954, respectively), indicating that they are not only metabolic executors but also critical “flow control points” in the network. They exhibit high strategy consistency across all three species, suggesting that maintaining the integrity of the core metabolic module is an evolutionary prerequisite for mammalian hibernation tolerance.

Cluster 2 (MCODE Score = 4.0) focuses on RNA processing and spliceosome machinery (global KEGG enrichment FDR < 0.05, Supplementary Table S3). This module is centered on SRSF3, DDX5, and FUS. Integrating the previously mentioned Stability Index (SI) analysis, we found that core members of Cluster 2 generally exhibit high transcriptional stability during hibernation. For instance, HNRNPA2B1 (Stress = 1850) serves as a bottleneck node connecting transcriptional processing and stress response. The identification of this module strongly proves that hibernating animals do not simply shut down protein synthesis; rather, by gathering a specific set of splicing factors and transcriptional transporters, they utilize selective post-transcriptional retention at the molecular level to ensure translation priority for critical survival proteins under low body temperatures.

Cluster 3 (MCODE Score = 2.0), although smaller in node scale, features a functional core consisting of PLPP3, SGMS1, and DEGS1 with a high degree of systemic coupling in sphingolipid metabolism (global KEGG enrichment FDR < 0.01, Supplementary Table S3). This module highlights membrane cold adaptation as another critical aspect of hibernation physiology. In extreme cold environments, cell membranes are prone to thermodynamic phase transitions from a liquid-crystal state to a gel state, leading to membrane protein inactivation and the collapse of ionic homeostasis. Our data show that the pivotal nodes SGMS1 and DEGS1, by precisely regulating the dynamic ratio of ceramide to sphingomyelin, achieve thermodynamic stabilization of the biological membrane at the molecular level. This homeoviscous adaptation strategy is not a passive metabolic suppression but an active reconstruction of physical homeostasis. This reveals how the hibernating liver maintains structural integrity and signal transduction efficiency at temperatures near 0 degrees Celsius by altering membrane lipid composition.

### 3.5. Systemic Stress and Bottleneck Analysis Reveals Communication Security in the Hibernation Network

Through a deep audit of global network topological indicators, we found that besides having high betweenness centrality, ADCY6 and PLPP3 also exhibit Topological Coefficients significantly different from constants, reflecting their isolated coordination roles in heterogeneous networks.

Specifically, EGR1 (Stress = 1048) and SLC2A1 (Stress = 2216) demonstrate extremely high systemic stress values, meaning they are indispensable paths connecting different functional clusters in the network. Although the strategy of EGR1 belongs to Species-Specific Synergy, its “monopolistic” position in the topological structure indicates that even if different species choose different regulatory polarities for hibernation onset, their dependence on EGR1 as a topological node is universal. This “conserved architecture, divergent logic” pattern explains why the hibernation phenotype possesses high evolutionary robustness: the system ensures the uniqueness of signal transmission through high-stress bottlenecks while achieving adaptation to different ecological niches through species-specific synergistic logic.

## 4. Discussion

To accomplish our objective of characterizing transcriptional regulation, this study integrates multi-omics data from three representative hibernating mammals and characterizes cross-species hibernation regulation at a systems biology level. Our data not only confirm the conservation of key metabolic pathways but also reveal the kinetic characteristics of the deep decoupling between transcriptional synthesis and steady-state abundance by introducing the (SI) and network topology analysis.

### 4.1. EGR1 Kinetic Decoupling: Insight into Evolutionary Barriers and the “Molecular Firewall” of Hibernation Onset

The sharp contrast exhibited by EGR1 in this study, specifically the nascent transcriptional burst (Sq GRO-seq: 4.42) versus the steady-state downregulation (Chipmunk RNA: −4.34), suggests a strictly regulated biological process; rather, it profoundly reveals the evolutionary barrier for mammals entering a low-metabolic state [20]. Within the ground squirrel, while EGR1 shows a rapid induction in nascent transcription (log2FC_GRO = 4.42), its steady-state level remains nearly at baseline, leading to an extremely low Stability Index. This “high synthesis, zero accumulation” kinetic feature, combined with the steady-state plummet observed in the chipmunk (log2FC = −4.34), constitutes the molecular lock for hibernation onset.

Based on these observations, we propose a rapid-induction and rapid-clearance kinetic model in which EGR1, acting as an immediate early gene (IEG), triggers a transient transcriptional surge upon sensing hibernation induction signals to activate downstream programs. However, the system immediately initiates a “fast brake” form driven by extremely high turnover rates, acting as a rapid clearance mechanism to deplete mRNA stocks. This decoupling prevents unnecessary protein accumulation and proteotoxic stress caused by high-activity transcription factors during deep torpor [21]. This model is critical because translation is profoundly depressed during torpor, as evidenced by the widespread disaggregation of polysomes into monosomes [22]. This ability for “transient perception and immediate clearance” of immediate signals, potentially mediated by RNA-binding proteins (RBPs) that promote mRNA decay [23], constitutes a core evolutionary threshold distinguishing hibernating species from non-hibernators, thereby ensuring metabolic robustness in extreme environments [10]. This distinction is further emphasized by comparative studies showing that while Egr-1 is often non-specifically elevated during forced hypothermia or anesthesia in non-hibernating models, its expression remains tightly controlled or uniquely patterned during natural torpor [24]. Our discovery of a dramatic nascent transcriptional burst of EGR1 (log2FC = 4.42) in ground squirrels, immediately offset by rapid turnover, reveals that true hibernators have evolved this rapid transcript clearance mechanism to selectively harness the signaling power of EGR1 while preempting its potential proteotoxicity during deep torpor.

### 4.2. Transcriptional Kinetic Decoupling: A Precise Survival “Reserve” Strategy

For a long time, researchers have tended to equate changes in mRNA abundance directly with functional status. However, the extreme Stability Index performance of SLC2A1 (SI = 2.01) and EGR1 in this study provides strong evidence for the existence of widespread post-transcriptional regulation during hibernation. We believe this kinetic decoupling has significant physiological compensatory meaning. For example, the sudden drop in SLC2A1 (GLUT1) transcriptional activity reflects the cell’s active shutdown of glucose uptake, but its high retention of steady-state mRNA ensures that existing transcripts are available for rapid translation upon arousal [25]. This stabilization may involve the redistribution of RNA-binding proteins like PABP-1 to subnuclear structures or specific mRNA-protein interactions that protect transcripts from exonucleolytic cleavage despite low transcriptional output [4,23]. Furthermore, some transcripts may even undergo slow elongation and failed termination during torpor, ensuring they are “pre-set” for immediate processing once rewarming begins [6].

### 4.3. ACLY and CPT1A: Cross-Species Conserved Metabolic Engines

In network mining, Cluster 1, as the module with the highest MCODE score, is anchored by ACLY, CPT1A, and ACOX1. ACLY exhibited a rare Triple Conserved upregulation polarity in this study. Considering that ACLY is a key hub for transporting Acetyl-CoA from mitochondria to the cytoplasm, its consistent cross-species activation suggests that lipid remodeling, rather than simple energy consumption, is the common evolutionary cornerstone for mammalian cold tolerance, paralleling the shift toward fatty acid oxidation seen in other metabolic depression models [4,11,26]. Furthermore, CPT1A, as the rate-limiting enzyme for fatty acid beta-oxidation, showed a very high Stress score (708), indicating it carries a massive communication load systemically [27]. During evolution, while different species may exhibit synergy in minor metabolic steps, the system ensures the robustness of the switch from carbohydrate to lipid utilization by locking the CPT1A topological bottleneck [28].

### 4.4. Signaling Hubs: System Commanders of the Hibernation Phenotype

Betweenness centrality analysis pushed ADCY6 (BC = 0.3356) to the top of the regulatory network. The cAMP signaling pathway involving ADCY6 appears as a high-weight topological hub in multiple species. Traditional differential analyses often overlook ADCY6 because its log2FC change is not as dramatic as certain metabolic enzymes. However, from a systems topology perspective, its “low amplitude, high betweenness” characteristic makes it an ideal steady-state regulator, acting like a traffic controller to finely dispatch signal flows across the system.

The identification of ADCY6 as a central hub is supported by biochemical evidence that adenylate cyclase (AC) retains significant functionality (approximately 25%) even at 4 °C [29]. This cold resistance is likely facilitated by hibernation-associated changes in liver plasma-membrane lipid symmetry, which shift the temperature threshold for enzyme activity and maintain signaling integrity at near-freezing temperatures [30]. We observed that many signaling genes belong to ‘Species-Specific Synergy,’ reflecting a subtle evolutionary arrangement: the regulatory architecture (topology) is conserved, but the regulatory logic (polarity) is plastic. This flexibility explains how the ground squirrel, chipmunk, and hamster evolve divergent physiological limits while maintaining basically the same biochemical foundation for hibernation.

Beyond evolutionary biology, our identification of SLC2A1 (GLUT1) as a stability-compensated factor mirrors the glucose-sensing forms observed in clinical “myocardial hibernation” [31]. This adaptive logic also aligns with the metabolic reprogramming induced by SGLT2 inhibitors, which elicit “aestivation-like” energy conservation patterns to protect renal and cardiac functions [26,32]. These parallels suggest that hibernation-inspired transcriptional kinetics may provide a brand-new molecular blueprint for metabolic therapy.

**Figure 3:**
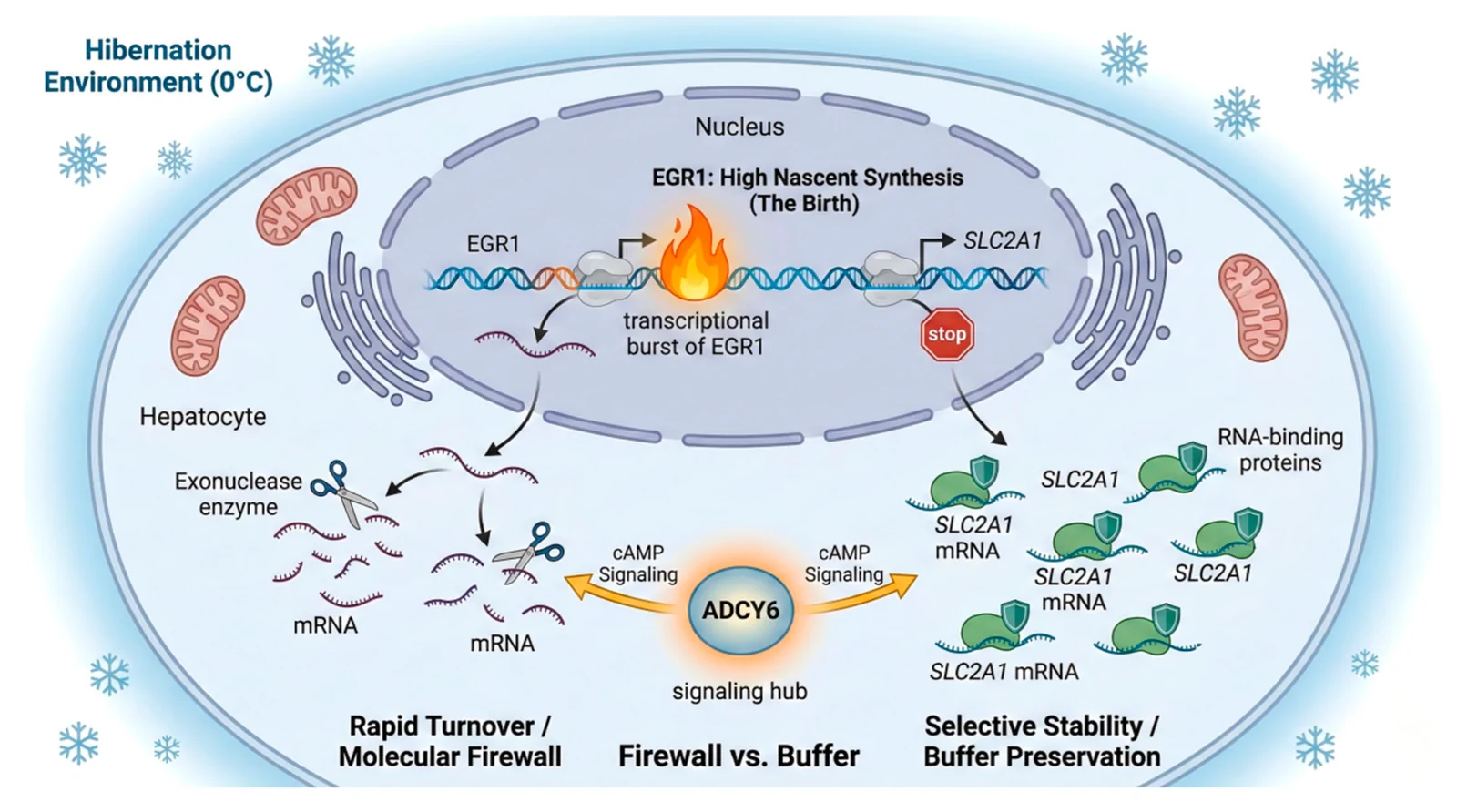
The “Firewall and Buffer” model of evolutionary robustness in hibernation. A conceptual model illustrating the dual-kinetic strategies of the hibernating hepatocyte under extreme cold (0 °C).

In summary, our multi-species integration and kinetic modeling transcend traditional steady-state descriptions by defining a dual-track regulatory landscape: a ‘Molecular Firewall’ for immediate response and a ‘Buffer Preservation’ for structural resilience (Figure 3). This unified model provides a molecular blueprint for understanding the evolutionary constraints and adaptive plasticity that govern mammalian metabolic robustness. Furthermore, by distinguishing between synthesis and degradation, our framework addresses critical methodological concerns regarding transcript stability and abundance that can be confounded by tissue handling or sampling protocols in post-mortem studies [33].

### 4.5. Limitations and Future Directions

While our multi-species integration provides novel insights into hibernation kinetics, there are still several limitations. First, due to the technical challenges of capturing nascent transcription in vivo, GRO-seq data was only available for the 13-lined ground squirrel. Consequently, the kinetic decoupling patterns (e.g., the SI values) observed in the ground squirrel currently lack direct cross-validation in the chipmunk and hamster at the nascent RNA level. Second, the modest sample sizes in our transcriptomic datasets (*n* = 3 to 5 biological replicates per group) may limit statistical power, potentially leading to the under-detection of genes with subtle expression changes, although our rigorous application of the DESeq2 pipeline and FDR correction helps to mitigate false positives. Furthermore, our study relies on bulk liver tissue, meaning that alternative explanations such as slight differences in sampling time points across species, potential technical biases inherent to cross-omics comparisons (e.g., GRO-seq vs. RNA-seq quantification), and hibernation-induced changes in liver cell-type composition cannot be entirely ruled out.

Future investigations should prioritize single-cell transcriptomic profiling (scRNA-seq) to deconvolve tissue-level heterogeneity during torpor. Additionally, experimental in vivo knock-down models or pharmacological interventions will be necessary to functionally validate the regulatory roles of topological hubs like ADCY6 and to transition our proposed kinetic models from observational hypotheses to established mechanistic pathways.

## 5. Conclusions

The findings of this study provide insights into how hibernating mammals actively manage transcriptional stress, challenging the traditional view of hibernation as a passive state. This study, through an integrated omics profiling of the 13-lined ground squirrel, Siberian chipmunk, and Syrian hamster, demonstrates that mammalian hibernation is by no means a passive genome-wide silence, but a precisely guided process of kinetic remodeling led by systemic stress hubs. We identified a cross-species conserved metabolic backbone supported by ACLY and ATF4, and for the first time in the field of hibernation evolution, revealed the compensatory form of “transcriptional burst–steady-state decoupling” through the SI.

Furthermore, the identification of topological bottleneck nodes centered on ADCY6 (BC = 0.3356) suggests an evolutionary pattern where core network topology is conserved, but regulatory polarity remains flexible. The system ensures metabolic robustness by locking core network architectures while allowing the regulatory polarities (Synergy) of local nodes to evolve flexibly across different species to adapt to diverse ecological niches. This finding breaks through the limitations of traditional steady-state transcriptomes in understanding hypometabolic condition, proving that post-transcriptional kinetic control—rather than simple transcriptional initiation—is the key threshold for organisms to cross evolutionary barriers and enter deep metabolic suppression. This not only provides a new perspective for understanding extreme survival strategies in nature but also establishes a powerful rodent model for decoding the molecular switches that govern metabolic suppression. These insights into evolutionary adaptations provide potential targets for translational biomedicine aimed at inducing hypometabolic states. Future work should prioritize single-cell transcriptomic profiling and functional in vivo knockdown models to definitively validate the physiological roles of these identified kinetic hubs.

## Data Availability

The original contributions presented in this study are included in the article and Supplementary File. Further inquiries can be directed to the corresponding author.

## Supplementary Materials

The following supporting information can be downloaded at https://www.mdpi.com/article/10.3390/vetsci13101062/s1.
