# Supplementary material for: Integration of Nascent Transcriptomes and Cross-Species Atlases Reveals Evolutionary Barriers in Transcriptional Kinetics During Mammalian Hibernation
Source: Vet Sci. 2026 Oct 9;13(10):1062. doi: 10.3390/vetsci13101062 (PMC13709684; doi:10.3390/vetsci13101062)
Supplement: Supplementary file 1 [file vetsci-13-01062-s001.zip › Supplementary_Figure S1_Genes_Heatmap(1).pdf]

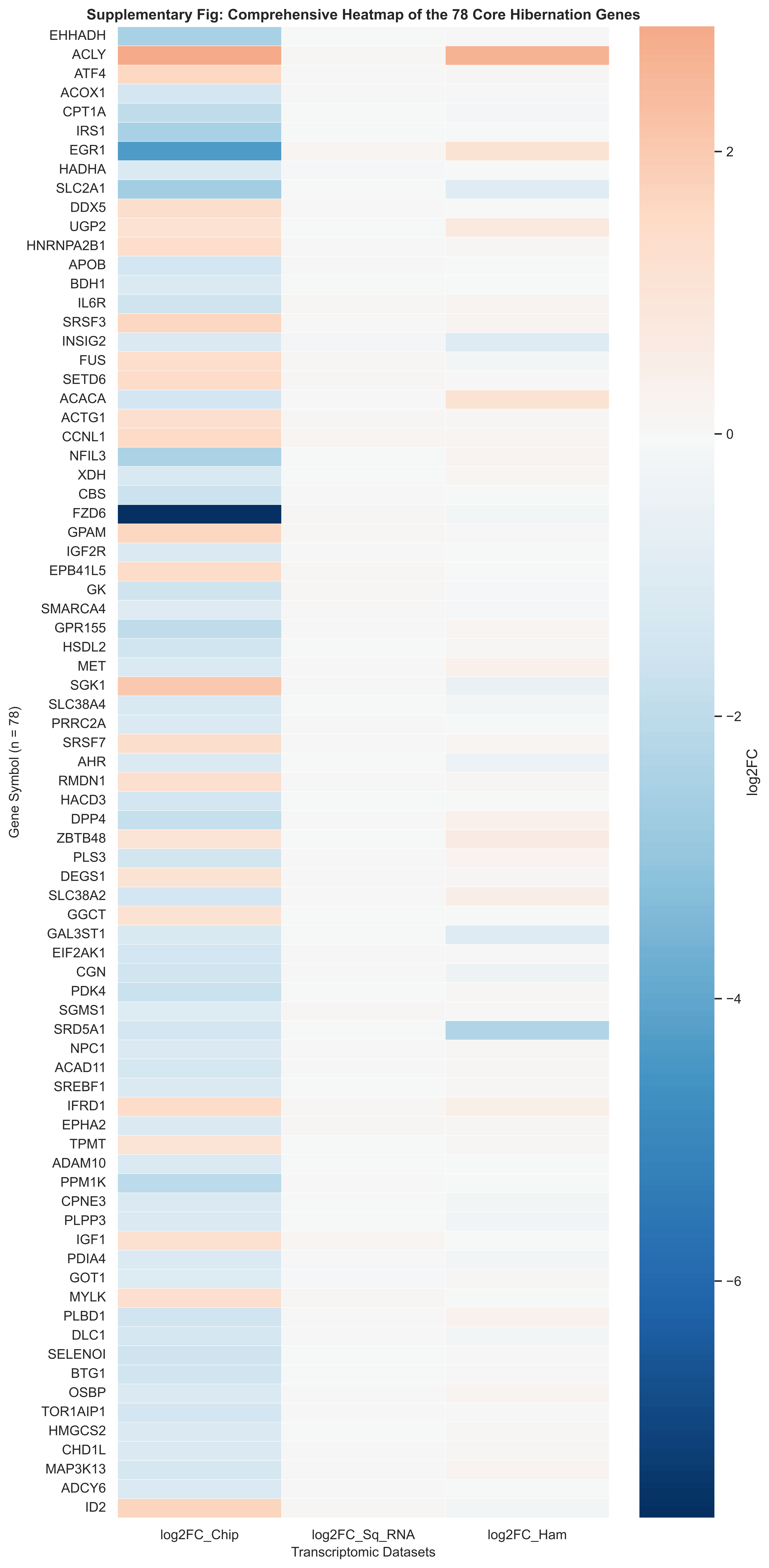

Supplementary Fig: Comprehensive Heatmap of the 78 Core Hibernation Genes
EHHADH
ACLY
ATF4
ACOX1
CPT1A
IRS1
EGR1
HADHA
SLC2A1
DDX5
UGP2
HNRNPA2B1
APOB
BDH1
IL6R
SRSF3
INSIG2
FUS
SETD6
ACACA
ACTG1
CCNL1
NFIL3
XDH
CBS
FZD6
GPAM
IGF2R
EPB41L5
GK
SMARCA4
GPR155
HSDL2
MET
SGK1
SLC38A4
PRRC2A
SRSF7
AHR
RMDN1
HACD3
DPP4
ZBTB48
PLS3
DEGS1
SLC38A2
GGCT
GAL3ST1
EIF2AK1
CGN
PDK4
SGMS1
SRD5A1
NPC1
ACAD11
SREBF1
IFRD1
EPHA2
TPMT
ADAM10
PPM1K
CPNE3
PLPP3
IGF1
PDIA4
GOT1
MYLK
PLBD1
DLC1
SELENOI
BTG1
OSBP
TOR1AIP1
HMGCS2
CHD1L
MAP3K13
ADCY6
ID2
Gene Symbol (n = 78)
log2FC_Chip
log2FC_Sq_RNA
log2FC_Ham
Transcriptomic Datasets
2
0
−2
−4
−6
log2FC
